# Exploring quality of life among the elderly in Hai Duong province, Vietnam: a rural–urban dialogue

**DOI:** 10.3402/gha.v5i0.18874

**Published:** 2012-12-22

**Authors:** Nguyen Thanh Huong, Le Thi Hai Ha, Nguyen Thai Quynh Chi, Peter S. Hill, Tara Walton

**Affiliations:** 1 Department of Health Policy, Faculty of Social Sciences, Behaviour and Health Education, Hanoi School of Public Health, Hanoi, Vietnam; 2 Department of Health Sociology, Faculty of Social Sciences, Behaviour and Health Education, Hanoi School of Public Health, Hanoi, Vietnam; 3 School of Population Health, The University of Queensland, Brisbane, Australia; 4 Masters of Public Health Candidate (Global Health Stream), Faculty of Health Sciences, Simon Fraser University, Burnaby, Canada

**Keywords:** quality of life, elderly, perception, qualitative study, Vietnam

## Abstract

**Background:**

Quality of life (QoL) is an important health index for the elderly, necessary for assessing interventions, and prioritising medical and social care needs. As the ageing population in Vietnam continues to increase, understanding important dimensions of QoL for the elderly is essential. There is a paucity of research in this area, however, and the available literature focuses on functional capacities. The purpose of this article is to explore perceptions on the dimensions of QoL among the elderly in Vietnam, to use these perceptions to broaden the concept, and to explore similarities and differences between those living in urban compared to rural areas.

**Method:**

Qualitative methods included in-depth interviews (IDI) with experts in ageing and elderly persons, as well as focus group discussions (FGDs) in three communes in Hai Duong province. IDIs and FGDs were recorded and transcribed. NVivo software was used to analyse the data.

**Results:**

Thematic analysis identified physical, psychological, social, environmental, religious, and economic as important dimensions of QoL. For elderly participants in both urban and rural areas, physical health, social relations, finances and economics, the physical and social environment, and psychological health were reported as important. Rural participants also identified religious practice as an important dimension of QoL. In terms of relationships, the elderly in urban areas prioritised those with their children, while the elderly in rural areas focussed their concerns on community relationships and economic conditions.

**Conclusion:**

Isolating individual factors that contribute to QoL among the elderly is difficult given the inter-relations and rich cross-linkages between themes. Elderly participants in urban and rural areas broadly shared perspectives on the themes identified, in particular social relationships, but their experiences diverged around issues surrounding finances and economics, their respective physical and social environments, and the contribution of religious practice. The study findings may help provide guidance for the development of a socially and culturally relevant instrument for measuring QoL among the elderly in Vietnam. The results will also be useful for developing policies and interventions that are responsive to the needs of the elderly, and reflect the themes perceived to be important.

Vietnam is in the process of social and economic transition. Social and political change has been profound, opening up the country's socialist economy through the process of *đ*
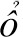
*i mới* (innovation), and moving away from socialist collectivism towards private enterprise and a market economy. The changes are reflected not only in their rising Gross Domestic Product, but also in almost all aspects of social and economic life (1, 2). With its recent economic growth and effective primary health coverage, life expectancy has increased from 66.5 in 1999 to 72.8 in 2009 (3). People aged over 60 years currently represent around 9% of the total population and are predicted to increase to 13.3% by 2,024 (4, 5).

**Policy Recommendations**

In order to improve the lives of ageing people in Vietnam there is a pressing need to develop comprehensive policies that cover the aspects identified as important to QoL. The following points summarise the key policy recommendations for the elderly drawn from this study:

- National policy promotes the development of inte-grated services by setting out a clear vision with the goals of maximising ageing people's QoL.
- Involvement of older people should be modelled in the way in which policy is developed, monitored, and evaluated. National policy supports integrated approaches that are centred on older people in the way in which they are planned and delivered, and in their quality. This involves supporting innovative approaches that offer choice, flexibility, and control by older people.
- Improving service system efficiency for service users with complex needs by taking a ‘whole-system’ approach, where services recognise their interdependencies, plan together to provide a comprehensive range of services, establish clear links between these services, and provide ways of tailoring services to local older population.
- Resource allocation supports the development of balanced service systems and is not directed to acute health care at the expense of prevention, comprehensive primary, and community services.
- Additionally, it is necessary to have prioritised policies focussing on the different needs of the elderly, such as improving social activities and connection for urban older people while creating mechanisms to provide economic stability for the rural aged.

However, this growth and associated urbanisation has also been associated with an increase in inequity, with a widening rural–urban income gap, as well as growing disparities related to geographic, social, ethnic, and linguistic isolation (6). Urban migration has been one response to these changes, offering opportunities to redistribute income between rural and urban areas (7, 8). On the other hand, however, migration has left the elderly isolated in rural communities without their traditional family support, as the younger generation seeks to take advantage of new opportunities. Family structures have altered, with shifts in the patterns of interdependence, and the elderly face many challenges in the transition because of changes in social structure and value systems, family structures, and living arrangements (8).

The negative synergy between an ageing population and an increasing burden of non-communicable disease has major implications for the health system, for the economy and for the QoL of the aged themselves. Previous studies have examined health needs, utilisation of health services and counselling for the aged, and the influence of lifestyle factors on health in ageing (9–12). While in urban centres, aged care facilities are now being promoted as an innovative alternative to direct support from the family, options for the elderly living in rural areas are limited, with most currently unable to access health care insurance, and dependent on basic state primary health care facilities (12).

QoL is an important health index for the elderly in every country, playing a key role in assessing interventions, and establishing essential medical and social care needs for the ageing population. Building on the recognition by the World Health Organization (13) that health is not merely the absence of disease but the ‘state of complete physical, mental, and social well-being’, international efforts over the past three decades have led to the development of multiple scales combining objective and subjective elements to measure functional capacity, broader health status, psychological well-being, social support, and the broader concept of QoL (14, 15). With increasing aged populations in most developed countries, understanding QoL of the aged has become a particular area of interest. However, research in the Vietnamese context is currently lacking, though the traditional sayings included in the headings of this article suggest that there is a long history of observation of issues of ageing, relationships, and perspectives on living that inform local debate.

Although the sense of psychological well-being is increasingly seen as influencing self-perception of health, much of the existing research on QoL among the Vietnamese elderly has focussed on the contribution of functional capacity to health. More recently however, there has been some emerging literature addressing the broader concepts of QoL among the elderly in Vietnam (16–19). Exploring socio-economic determinants of health, two studies conducted in 2010 examined the association between health-related quality of life (HRQoL) and socio-economic factors among elderly rural populations in Vietnam (16, 17). While these studies used different tools to assess QoL, the results were similar, with both finding inequalities in health status and QoL, specifically noting age, being female, and poor household economic status as being determinants of poor HRQoL. To gain a cross-cultural understanding of QoL, two studies conducted in 2011 compared HRQoL/QoL among the elderly living in rural areas in Vietnam with those in rural areas of Bangladesh (16) and Indonesia (18). Both found significant differences in HRQoL/QoL of the elderly between countries and concluded that socio-cultural differences were likely to account for these. While the results of these recent studies underscore the role of culture and socio-ecological context in influencing health and QoL among the elderly, a deeper understanding of the dimensions of QoL as a function of the local social and cultural realities specific to Vietnam is still needed.

This article describes research undertaken to explore the important dimensions of QoL that reflect the culture, life-experience, and views of the rural and urban elderly in Hai Duong province, Vietnam.

## Methods

The study was conducted in Hai Duong province about 60 km Northeast of Hanoi. The province has both urban and rural districts, with rural mountainous areas comprising 11% of the province. Socio-economic development and urbanisation processes are similar to that of other Red River Delta provinces in the North of Vietnam (20).

The study employed qualitative methods to explore and compare the dimensions of QoL for urban and rural dwelling elderly Vietnamese. In the first stage, key informant IDIs were undertaken with five Hanoi-based experts in ageing to seek their opinions on the QoL for the aged. Experts included sociologists, geriatricians, demographers, and sexual and psychological counselors for the elderly who had been identified during the literature review process, and were then contacted by email or phone to invite them to participate. During the interview, participants were asked to discuss their knowledge and experience of QoL research in Vietnam, and other countries, as well as the dimensions they perceived to be important and the factors that may serve to influence them. The aim of the key informant interviews was to obtain the experts’ experience about QoL so that these could later be used for triangulating the findings with the experiences of the elderly collected during the second stage.

In the second stage, IDIs were conducted with male and female elderly participants, who were purposively selected using a maximum variation sample (retirees/farmer/informal sector, urban–rural). Mixed gender FGDs were conducted concurrently to broadly explore the general themes identified from the literature and expert IDIs, while the aim of the elderly IDIs was to explore in greater depth and specificity more sensitive issues such as sexuality, finance, and social relationships. Eligibility for both the FGDs and IDIs required participants to be over age 60 years, physically and mentally capable of participating in an approximately one and a half hour interview.

Heads of the Elderly People's Union were contacted by phone, provided with the eligibility criteria, and then asked to identify potential participants. Eligible individuals were then asked by Heads of the Elderly People's Union to attend a meeting, where they were provided with information about the study, and invited to participate in either an IDI or a FGD.

Three communes were selected representing urban, rural, and rural–mountainous areas of Hai Duong, and two IDIs, and three FGDs (with 8–10 male and female aged persons per group) were conducted in each, a total of six IDIs and nine FGDs overall. As some themes included areas that were at times controversial – particularly in relation to sexuality – use of both IDIs and FGDs provided a mechanism for triangulating the findings. FGDs and IDIs with elderly people were conducted in a conversational manner by experienced interviewers and were tape-recorded and transcribed in the Vietnamese language. The IDIs and the FGDs included similar questions, which asked the participants to discuss their perception and understanding of the term QoL, as well as its potential dimensions, they were also asked to discuss what would make life better or worse for elderly persons, and to compare how gender or location would affect how one values QoL.

Thematic analysis of the literature search and key informant IDIs provided key themes used in the development of the question guides for elderly IDIs and FGDs. Transcripts of the elderly IDIs and elderly FGDs were coded using NVivo software by a group of five experienced researchers, with emergent themes not previously flagged for discussion. Selected transcripts were independently re-coded by a second researcher, to ensure consistency between coders. Reconciliation of codes, including emergent themes, was agreed by consensus of the whole group before all transcripts were finally coded.

## Results

Thematic analysis revealed a set of six themes of QoL perceived to be most important for the elderly in Vietnam: physical health, social relationships, finance and economics, physical and social environment, psychological health, and religious practice. These themes along with the sub-themes identified as corresponding with them can be found in Table 1. Descriptions of each theme are provided below, and each of these begins with a Vietnamese idiom, expressions which we found to be consistent with the contents of the particular theme.

**Table 1:** QoL themes and sub-themes identified from research findings

No.	Themes	Sub-themes
I	Physical health	1. Body pains
		2. Ability to move around
		3. Tiredness
		4. Dependence on treatment
		5. Ability to work
		6. Ability to serve themselves
		7. Sleep/easy to sleep
		8. Ability to hear and see
		9. Ability to remember
		10. Ability to do housework
II	Social relationships	11. Support non-economics for others
		12. Support non-economics from children
		13. Spouse intimacy
		14. Family sentiment^a^
		15. Role in the family
		16. Community relationships^b^
		17. Relative relationships^b^
		18. Role in the community
		19. Participation in community's activities^b^
		20. Sexual activity^a^
III	Finance and economics	21. Stable income
		22. Economic support from children^b^
		23. Economic life is assured
		24. Economic support for others
		25. Economic dependence on children^b^
		26. Having favourite food to eat^b^
		27. Daily expenses^b^
		28. Expenses for community activities^b^
		29. Expenses for health care
		30. Adequate facilities^a^
IV	Physical and social environment	31. Physical environment
		32. Housing
		33. Social security
		34. Social service accessibility
		35. Information accessibility
		36. Health care service accessibility
V	Psychological health	37. Satisfaction of social relationships
		38. Feeling bored
		39. Satisfaction of family/children
		40. Being respected
		41. Not having anxiety about the after life
VI	Religious practice	42. Spiritual belief and religious practice

a Emphasised in urban

b emphasised in rural; the remaining are mentioned in both rural and urban.

### Physical health

Khôn đâu tới trẻ, khỏe đâu tới già: ‘Young people are never wise, older people never strong’

Informants considered physical health a higher priority than material assets, linking it to harmony within the family and a sense of purposive community engagement. Access to health services was perceived to be important for maintaining health.

Within urban FGDs, concerns for material needs were minimised, with health issues and access to services seen as more important. Hypertension, heart disease, diabetes, rheumatism, and hearing problems were cited as common, and hearing problems or joint pain presented obstacles to participation in the ‘morning exercise clubs’ – groups meeting in local parks for low impact calisthenics.

The significance of health was not limited to the physical, but *to be healthy is the most important, but not only physically, but also spiritually* (FGD 2_Urban). In one FGD an older man offered a poem, reminiscing on a life well spent, and the importance of a peaceful soul and a settled mind.

Rural FGDs supported the view of health as having enough to eat, sleeping well, and *to have a mind free from worry* (FGD 6_Rural). But health within the family also means harmony and the maintenance of tradition, important in a society still influenced by Confucian values^1^: *I care firstly about my family, secondly about keeping family tradition. The family tradition will affect my health: if there is no argument or conflict in the family, then I feel happy, and free from headaches* (IDI 4_Male_Rural).

Good health for the elderly was understood by informants to be purposive, the key to their continued usefulness and engagement in rural society. It allows them to remain part of the community, keeping their garden, picking up the grandchildren, and doing the housework. But these roles also come with prescriptive expectations, that their health will enable them to practice behaviours that are ‘a mirror for the younger generations’.

Key informant interviews with experts tended to reflect their focus on the clinical, with arthritis and muscle pain, prostate disease, nocturia, and bladder control identified as key issues affecting QoL. Problems with seeing and hearing were recognised as impacting on social interactions and compromising the confidence of the elderly in engaging in community activities. Experts acknowledged that disease and disability were often accepted by the elderly as a natural part of aging – and not seen to be negatively affecting life.

### Social relationships

Con chăm cha không bằng bà chăm ông: ‘The children can't care for their father like his wife can’

The analysis of the social dimension showed the largest disparity in opinion between the elderly informants in both urban and rural areas and experts. Within FGDs, in contrast to expert IDIs, discussion around the theme of social relationships revealed quite prescriptive marital and filial relationships, with clear expectations around roles, and pleasure found for the elderly in the successful lives of their children and in their sense of connectedness to their communities and ancestors. Elderly IDIs gave insight into the tensions between social expectations and the personal experience of individuals coping with the compromises demanded by social change, and in particular the impact of rural–urban migration of the more economically productive family members.

The issue of sexuality divided opinions. The experts – largely clinically oriented geriatricians – insisted that continuing sexual activity is a key element of QoL for the elderly. Urban and rural informants raised the issue in FGDs and in individual interviews, but not approvingly, arguing that the elderly should be ‘models’ for their families, putting those desires behind them and concentrating more on their relationships with their children. All groups saw these inter-generational relationships as crucial to QoL.

When urban participants in the research raised issues related to sexual activity in the elderly, it was to indicate the inappropriateness of the idea. In one case, the opposition mixed implicit concern around the appropriateness of a widowed parent's intention to re-marry a younger woman – with clear sexual implications – and the explicit financial risks to his children's inheritance, with them insisting that he reimburse them for the value of their shared home if he was to re-marry. In another, there are clear prescriptions for what is expected of the elderly – even by their siblings:

I have a brother in Hanoi who calls me continuously asking me to find him a woman who is hard-working and honest for him to marry. I advise him ‘You shouldn't get married again. Your children are all grown up, and you should be a mirror for them to look in and a strong shoulder for them to lean on. [Getting married] would only be to have someone to live with – you wouldn't be up to anything else’. (FGD 3_Urban)

In urban areas, the elderly described tensions between independent living and access and influence in their families’ lives. Older people indicated that they want to live close to their children but not necessarily together. Increasingly, they may live together but not eat together – keeping their own kitchen to accommodate different diets, and more independent living – though sharing meals reasonably regularly. Some multi-generational households persist, though less than in rural areas. The extent to which modern lifestyles challenge ‘traditional’ Vietnamese family values was more evident in urban areas or in rural households where adult children had emigrated to find employment. Respondents reported situations where elderly had to ‘fend for themselves’, where ill parents were not given the immediate attention they needed, where their opinions expressed in community forums were discounted as irrelevant. Most confronting was the refusal to accept the obligation to care for elderly parents:

In our day, people always cared for each other regardless of whether they were poor or rich, but now, they do not. They only feel responsible for their own immediate [nuclear] family, not thinking of their parents. I've heard someone tell their father: ‘I earn money to feed my wife and children. Did you have to feed your father when you were my age?’ (FGD 2_Urban)

At times, the urban elderly were seen to impose their more conservative values and attitudes on their families, explicitly expressing their concerns over the ‘Westernisation’ of children's and grandchildren's life styles. With increasing age, strong traditional desires for a son and grandson to continue the family line are often expressed. In one IDI, a grandparent expressed a desire for their children to break the national ‘two child policy’ in order to secure a male grandson: *I don't feel comfortable about my grandchildren. I have two grandchildren but both are girls* (IDI 2_Female_Urban).

The issue of sexual activity in the elderly was downplayed in rural interviews and discussions; if anything, this was considered more of an issue in urban areas, linked to a loss of culture:

In the city, their way of living seems to be westernised. In the rural, we think sexual life is not necessary at this age. What important now is how to live healthy and merry. Old people should live pure, should go to pagoda^2^ to pray for their children and grandchildren. (IDI 1_Male_Rural)

The relationships between elderly parents and their children and grandchildren were seen to be of prime importance for both urban and rural informants. Given the more limited cash economy in rural areas, there was less discussion of how this was expressed financially – more in terms of respect, physical assistance, the links to grandchildren and the need for contact between generations. With closer living more common in rural contexts, tensions in the relationships with sons or daughters-in-law were raised here. Financial independence was exceptional, as the rural elderly were less likely to have pensions, but *if their children are rich or they can earn more money to support their parents, the elderly can live comfortably (sướng)* (FGD 9_Rural).

But this comfort in living is dependent on harmony within the household, with harmonious relationships between elderly parents creating a positive environment for their children. In contrast, the changing socio-economic environment brings uncertainty – particularly where children migrate to urban centres and are open to the corrupting influences of modern life.

Now we are worried about of our children when they go out of the community to work or study. Social evils are present everywhere. Before, if my children travelled, I knew they would come back, but now, I cannot be sure. The society now is more complex than in our time. (IDI 6_Male_Rural)

Clearly psychological health is interconnected with social relationships, with the financial context for rural elderly dependent on their children an important influence:

My neighbour has four children: two of them are alcoholic, two have gone away to look for work, so my neighbour worries all the time about his children. He can't sleep at night and now he is very sick. (FGD 6_Rural)

Both rural and urban informants spoke of the importance of status in the community and family to their QoL – respect for the old is traditionally important – but increasingly eroded. The elderly valued being able to contribute to debates at home and in the community. Moreover, they expressed concern when young people no longer acknowledged them on the streets or valued their contributions to community meetings.

Despite the constraints that they experienced compared to their urban counterparts, the rural elderly saw their QoL enhanced by their ongoing social networks, their access to community activities such as morning exercise, singing or poetry clubs.

People in rural areas have a sense of neighbourhood. They can just drop-in and chat with their neighbour when they want to. Not like in the city: people close their doors to neighbours. They have no close relationships with their neighbours even though they live next to each other. That's why some people don't feel happy. (FGD 6_Rural)

The sense of community extends beyond their neighbours into a sense of community that extends back through generations. While pagodas tend to be more directly associated with formal religion and religious practice, community temples are erected around the memory of significant community ancestors, providing links through them for the communities that they have shaped, and integral to community identity: *Old men go to the community temple; old women go to the pagoda. There they talk – about their community culture and customs* (IDI 4_Male_Rural).

Expert interviews, however, based on their research and experience, tended to emphasise marital relationships over other communal relationships. For them, the importance of marital relationships lay in continued sexual engagement, a ‘natural’ part of life, though they conceded that this may not be popularly accepted:

If old people talk about love, or marriage or sex, then people will think they are depraved. I think we need to be more humane – our thinking should be more realistic. If old people have good health then they will have sexual desire. Sexual life is the most important aspect of quality of life. (IDI 2_Expert)

While experts saw the primary social relationship being between elderly marital partners, in common with other informants, they also saw their relationship with their children as significant, with the evidence for the quality of these relationships very much in the public eye:

Respect from children and society is very important for old people. We can actually measure this respect by counting how often the children call their parents or visit them each week, and their willingness to help when older people have problems. (IDI 2_Expert)

The expert panel considered that the increasing distance within families could be offset by relationships in the broader community and that this occurred with more ease in rural areas than in urban areas. In urban areas, even living closely may not ensure satisfying contact: *their children may go to work all day and their grandchildren are at school. In the evenings, their children go to English class and their grandchildren go out with friends, and they are still alone* (IDI 2_Expert). What they did acknowledge was that the elderly highly valued their right to express opinion or judgement and have it considered by family or community. They keenly sensed the perceived loss of respect for the opinions of the elderly.

### Finances and economics

Trẻ cậy cha già cậy con: ‘Children rely on their parents, older people rely on their children’

All groups consulted identified financial independence for the elderly as integral to their QoL, linking this theme to their happiness, their sense of security, their sense of independence, their ability to contribute to public debate and to engage in community life. Both urban and rural elderly expressed their fear of being sick and the need to go to hospital. For many, they do not have financial reserves, and costs are unpredictable – and involve more than just the direct costs. The differences between urban and rural are most acute here – with urban dwellers more frequently able to access government pensions, and rural elderly marginalised in the cash economy. While they contribute in non-financial ways to the economic status of households through child-care and household chores, these contributions are often not formally acknowledged.

Pensions were more likely to be received by the elderly who have worked in government positions in urban areas, and as a result, concerns around financial independence were less frequently raised in urban than in rural interviews:

Almost all the elderly in city have pension so they have relatively sufficient money for living. But almost elderly in the rural are farmers, they do not have pension and they have to live in poorer conditions than in the city. (FGD 2_Urban)

The economic opportunities for the elderly are clearly greater in the city, though in both urban and rural areas situations they make significant household contributions.

For rural respondents, the more constrained financial circumstances are expressed in quite concrete expressions of QoL. Financial dependence, and the lower prevalence of pension support, means the elderly continue to work long past prescribed retirement age. *Old people in the urban have pensions. They are happier (sướng) than us. We are farmers. If you ask each one in this room, we have the same life condition* (FGD 8_Rural).

Having not worked in paid employment, the rural elderly are ineligible for government health insurance. While the rural elderly do not benefit from pensions, the elderly who continue to farm are still liable for taxes and often need to pay additionally for work they are no longer capable of performing. Their QoL is largely determined by the financial standing of the family.

For both urban and rural, disposable finance is reflected in their diets: food is important culturally and the focus of social interactions. As would be expected in rural communities, the quality of food is clearly important, particularly for those rural informants with a long history in produce. Rural informants were acutely aware of the difference that marginal financial changes make: more money means better quality in food – meat at every meal, rather than occasionally. The lack of financial liquidity impacts in simple ways – such as the simple luxury of a snack to follow morning calisthenics:

In the city, after doing morning exercise, old people can have breakfast with noodles or something else. But old people in the rural areas don't have [can't afford] anything to eat for breakfast. (FGD 6_Rural)

But for some rural elderly, the solutions are simple: *I am happy when I have a glass of [herbal tonic] wine to drink every day. If you drink it regularly it keeps you healthy* (FGD 9_Rural).

### Physical and social environment

Ði hỏi già v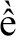 nhà hỏi trẻ: ‘Ask your elders before going out; ask children when you come back home’

The physical and social environments reflected concerns around safety – both in terms of the physical environment itself – with its noise, pollution and lack of comfort – and social changes towards more nuclear family living spaces, with less communal interaction. For respondents in rural IDIs and FGDs, there was a greater emphasis on the physical environment, both in terms of increasing risks, and in terms of comfort for living. Interestingly, the social environment issues were mentioned more by urban than by rural, with urban living increasingly globalised, with both Western, but also Japanese and Korean cultures influencing changes in fashion, lifestyle, media and diet. For them, the rapidly changing social environment presented a challenge in terms of locating themselves meaningfully.

In urban centres, the environment in which the elderly now find themselves was the focus of a number of anxieties, with industrialised centres a particular concern. The cost and quality of fruit/vegetable reflected uncertainty of the origin of foods, and concerns about the increased use of pesticides in foods purchased in urban markets were reported by urban respondents and listed as a risk of urban living by their rural counterparts. But uncertainty regarding the influences on the city was not limited to the quality of air, food and water – the social and physical environments interact in ways that can be intimidating for the elderly:

The social and cultural environment is polluted now. I went out and saw not only my grandchildren at home but also many younger people in the street have blue and red hair. I asked them why they do that and they told me I am a ‘backward man’. I feel broken hearted for that. (FGD 1_Urban)

Elderly respondents vocalised their ambivalence around current social trends expressing strong concerns around the influence of the media, which promotes the desire for increasingly consumerist lifestyles. Their persisting socialist commitment contrasts with the more individualist orientation of their grandchildren. For a generation which has lived through post-war austerity, there is concern that the media promotes these ‘modern’ lifestyles without building skills to achieve and sustain them:

The TV doesn't teach young people how to make themselves prosperous—it only shows fighting, dancing and hip-hop and travelling. It shows them how to make themselves seem beautiful, but not how to earn the money so that they can afford beautiful clothes and travel. They learn how to spend a fortune, but not how to make their fortune. In our time, our parents taught us practical things: how to work properly in the fields, how to reduce the weeds for better crops. (FGD 2_Urban)

Some of the elderly, however, recognised that this transformation is grounded in socio-economic changes that the young are better prepared for, and that personal hope for the future has to be linked to confidence in young people: *I believe in young people. They have different awareness of politics and culture and they have the knowledge to solve the everyday problems we are facing* (IDI 1_Male_Urban).

Rural respondents were acutely aware that their environment had advantages, in particular air quality and the relative quietness compared to urban centres. There is recognition by rural elderly of the material benefits of city life, but the growing economy and remissions from migrant family workers means that much of the advantages of the city are now accessible in the country:

If you compare our place with the city, of course the city is better than here. However, I come to the city many times and I see that some places in the city are very poor. Honestly I think those places are not as good as in the rural area. Here we also have television, refrigerator, and etc. We have fish, pork, beef, and fresh fruit. I think it's even better than the city! (FGD 4_Rural)

As in the cities, however, increasing industrialisation has brought increasing risk – often associated with insecticide contamination of food or water, though sometimes the threats of rural life, real or perceived, persist.

Before, we weren't sick so often. Today, because we are sick so frequently, we have to avoid many things. We used to prepare tea with rain water, it was delicious, but now it tastes tart. The living conditions in rural areas are not comfortable for old people. The bathroom and toilet is not in the house—if they wake during the night, they risk getting sick because of the strong winds outside or even snake bite. (IDI 5_Male_Rural)

The expert group also expressed a similar view on the advantages and disadvantages of physical and social environment on both rural and urban elderly QoL.

### Psychological health

Gùng càng già càng cay: ‘The older the ginger, the spicier it is’

Constructs around psychological health exposed the impact of rapid social and economic change on the worldview of the elderly interviewed for this study, and their need for a sense of continued relevance and usefulness in society. The erosion of traditional values concerned some, though the centrality of ‘*sướng’* – happiness or contentedness – to their lives, reflected the persistence of deeper values.

In urban centres, the elderly were distressed that their participation in community activities was compromised because of perceptions that their opinions were not seen as relevant. Within local Communist Party Cells, they felt that the opinions of retired members – many of whom had long associations with the political struggle for Vietnam – were perceived to lack currency or value, and were seen as ‘out of touch’. In part this reflected a shift in ideological values, accentuated since the introduction of ‘*đ*
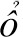
*i mới’* (innovation):

Lifestyles now are affected by the influence of the West. Young people don't maintain traditions, and the elderly worry about their families and society and worry that they will be abandoned. (FGD 3_Urban)

Rural informants similarly acknowledged that psychological health depended on feeling ‘useful’ and that the current economic situation offered greater opportunities for an active role than ever before. In part, this window of opportunity is attributed to political change with individual efforts contributing directly to family wealth, rather than being absorbed through communal obligations to a state based collective.

However, family was seen as the key to psychological contentment in terms of living positively and securing the future economically. ‘*Sướng’* was used often by the elderly to describe this happiness, though the word is more complex than happy, and implies a sense of completeness, of shared joy, of all aspects of life being ‘right’.

Older people should think about their children when they do anything. When the family and their children's family are happy, this is what the older people want to see the most. This will make them feel at ease (sướng). (IDI 5_Male_Rural)

This *suong* is reflected in the consensus from all groups that *the most important is a mind free from worry* (FGD 6_Rural). The ‘worry’ reiterated in the statements by experts and both elderly groups certainly included the need for basic needs to be met but emphasised health and evidence of success within the family. Psychological health is not considered an individual attribute, but a shared one, with obligations for the elderly to be models for others in terms of prosperity, but more importantly, harmony:

In general I am satisfied with my life. My family is not rich, but my children are good: they do nothing to make us sad. Not like some families that are rich, but their children cause them headaches! (IDI 5_Male_Rural)

Surprisingly, expert opinion was divided on psychological health. While the need for freedom from worry and care was broadly accepted, some experts challenged the idealised view of the ‘simple life’ of the elderly, making the point that minimal needs must be met before contentment can be considered:

A mind free from worry is very important to old people. If they have a good material life they can then think about entertainment, but if they are living in a poor rural area, the material and spiritual are linked. (IDI 3_Expert)

### Religious practice

Trẻ vui nhà, già vui chùa: ‘Young people are happy at home; the elderly are happy at the pagoda’

The theme of religious practice placed emphasis on spiritual practice and personal contentment, shared with the previous themes of psychological health and social relationships. While religious practice was not raised as an issue in expert panel discussions, and only twice in references from urban focus informants, it was spontaneously offered in rural elderly focus groups.

In urban FGDs, going to the pagoda was associated with feelings of contentment, of relief. In traditional thinking for both urban and rural informants, age frees people from their responsibilities and pre-occupations in the home to spend more time in the pagoda, reflecting more on the spiritual aspects of life.

In the rural groups interviewed, spiritual practice was emphasised as important to the essential identity of the elderly. Religious practice offers psychological as well as spiritual benefits. The temple gives a sense of community to older men, a connectedness to their past. While it does have a religious dimension, the communal temple is a social space, a meeting place particularly for older men that also connects them with the history of their community or profession, through reverence offered to their ‘ancestors’. Regular pagoda attendance or church attendance was seen to provide this social contact, but also to offer other transformative benefits. Religious practice was described as having an impact on the elderly themselves, but also transforming lives of others.

For rural respondents – both Buddhist and Roman Catholic – righteous living brings health for themselves and benefits for their families: *People need to love each other and those more vulnerable than they are …. If we live well now, the future of our children will be better. If the father eats salt, the children will be thirsty* (FGD 4_Rural).

For those informants committed to religion, purity of thought and of intent was integral to their discussion of its transforming impact, often defined in terms of the surrender of sexual desire consistent with popular expectation: *You need to be pure when going to the pagoda. Pure means that you need to give up your sexual desire: it is not important to old people* (IDI 3_Female_Rural).

## Discussion

Using a qualitative approach in the current study, we analysed and triangulated narratives from experts and elderly people living in both urban and rural areas to get an in-depth understanding of the meaning of QoL and to identify the dimensions of QoL that are of importance and relevant for older people in Vietnam. The results from this study reveal six key themes that are common among elderly people in both rural and urban Vietnam when talking about their QoL: physical health, social relationships, finance and economics, physical and social environment, psychological health and religious practice. The findings of our study identified similar themes to a study conducted in rural Bangladesh by Nilsson and colleagues (14), though the detailed expression of these was clearly shaped by local social, cultural and economic factors. Thematic analysis of the data demonstrated the difficulty in isolating factors contributing to QoL, and the complex interdependence of the themes. Rich cross-linkages were apparent among psychological health, social relationships, and finance and economics. Despite this, two issues relating to QoL appear to dominate the responses of both rural and urban respondents: finances and economics, and social relationships.

Financial security appears to be a key concern in a state moving from a socialist economy to a mixed economy under *đ*
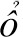
*i mới*, with the loss of the previous protection afforded by socialist structures. The economic impact of *đ*
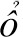
*i mới* was also apparent in the lived experience of the elderly, with urban retirees from (usually State) salaried positions entitled to a State pension, but rural elderly not eligible for these benefits.

Urban elderly report greater access to funding and a higher potential for continued economic activity than their rural counterparts, who suffer the limitations of a subsistence economy, with fewer options for activities, and no financial resources to take advantage of them (8). The inverse relationship between socio-economic status and QoL is well known and has been confirmed in recent comparative studies among the elderly populations of Vietnam and Indonesia as well as Bangladesh (16, 18). Urban migration provides some limited compensation – with the younger generation returning remittances from their urban employment – but at the cost of isolation and familial fragmentation for the rural elderly.

In the accounts of our respondents, rural–urban migration represents a cost that is acutely felt in terms of the second most significant issue for QoL: social relationships. For all informants, it was clear that healthy social relationships – marital, filial, and communal – were crucial to creating the sense of *suong* – completeness, contentedness – on which QoL for the elderly depended. However, the area of social relationships also produced the most contested issues, particularly in dealing with sexual activity in the aged. While experts valued sexual relationships as a major contributor to QoL, this issue did not emerge in discussion with both rural and urban informants. The tension between prescriptive traditional roles and more liberal trends is evident in the examples offered in the FGDs and supported in IDI. While overt sexual interest was seen as inappropriate to the elderly, and in some way clouded the ‘mirror’ the elderly were meant to hold up to the younger generation, all acknowledged the primacy of marital relationships to QoL. The proverb *Con chăm cha không bằng bà chăm ông: ‘The children can't care for their father like his wife can’*, suggests that a discreet sexual relation may be implicit in the superior care a wife offers her husband, though this may not be overtly acknowledged. Although there are similar between the perception of elderly and expert group, the discord between these two participants groups clearly exists on social relationship theme. This difference can be explained by strongly influence of Confucian philosophy, social structure, the ways Vietnamese elderly express emotion and somatisation tendency.

The importance of familial relationships, however, remained critical to QoL among both rural and urban participants, with the elderly needing to be both valued by their children, and dependent on their children's happiness and prosperity for their own. Urban and rural informants saw differences between their physical and social environments, their perceptions shaped by their own experience; in finances and economics, inequities between advantaged urban elderly and rural elderly with less access to a cash economy were marked. However, the commonalities in the area of psychological health and social relationships suggest a shared deep valuing of links with family and community. Having a link and role in the family and the community is also emphasised as the most important aspect of QoL of elderly people in rural Bangladesh (21). The elderly's concerns around having a ‘voice’ within the decision making frameworks of their own extended families was also reflected in their concerns around their place within the broader social framework, with rapid social and economic change seeming to easily marginalise them in terms of their social and economic contributions. Despite these concerns, there is sense in which the elderly continue to play a stabilising and supportive role within families, and that they are negotiating their way into new relationships in a rapidly changing world, and that their capacity to maintain their QoL is intimately linked to this. Traditional Vietnamese culture exerts a positive influence on elderly people by providing guidance in dealing with the process of ageing.

Although considerable attention has recently been paid to the elderly in Vietnam, the vast majority of this attention and related research has focussed primarily on the physical health of this population. This study has examined older people's perception of QoL and contributes to increasing and ongoing efforts to better understand the dynamics of ageing. This study also indicates that interdisciplinary studies should be conducted to assess the needs of the diverse elderly population with respect to specific needs related not only health services but also social services, in order to develop policies and identify specific programs at different settings.

Given the importance of QoL among the elderly, it is not surprising that there has been increased attention in assessing this variable, with general instruments validated in ageing populations, as well as the development of specific instruments for the aged (21). However, the search for culturally compatible instruments that maintain links with international understandings of QoL while acknowledging local social and cultural realities, places researchers in a difficult position of deciding whether to adapt existing scales or to develop independent instruments, with experience from Bangladesh, India, Lebanon, Taiwan, and Thailand presenting a range of approaches and solutions (15, 22–27). Consequently, the findings from this study may prove useful in guiding the adaption of an existing measure, or, alternatively, in the development of a new culturally relevant tool, specific to the Vietnamese context.

Additionally, this study was undertaken to raise awareness to the issues surrounding QoL of this population and create appropriate changes within elderly care. The results of this study have a number of important policy implications. First, the responses from the elderly participants provide a first-hand account of the multiple dimensions of QoL that are perceived to be most important among this population, perspectives which are invaluable for developing appropriate interventions that are responsive to the needs of this population. The identified issues and implications allows institutional policy makers, administrators, and program planners to better understand the needs of this population, undertake informed policy and planning decisions, and pursue funding allocation in an effort to improve the QoL of the elderly. It is especially important to recognise and address the needs of the ageing population that is projected to increase, making it relevant and pertinent for ongoing gerontological research. Moreover, this information enables participation of the elderly in the policy process, providing them with the chance to actively contribute to society and that will have a benefit on the QoL of the rest of their life. Secondly, the interconnectedness of multiple dimensions that were identified in this study highlight the importance of considering QoL from a broader perspective, and the need to develop comprehensive public health policies that not only encompass the functional capacity, and physical aspects of health but also incorporate psychological, social, environmental, and economical aspects. More importantly, these findings emphasise the need for an intersectoral approach to developing policies for improving the lives of the ageing population in Vietnam. For these public policies, the issues at stake are not only to undertake appropriate changes in the parameters of the welfare system but also to reconsider the balance between public and private initiatives, and notably to thoroughly analyse to what extent future necessary adjustments can be achieved through the play of market forces and to what extent policy intervention is required.

## Strengths and limitations

One of the strengths of this study was that the collection of qualitative data provided an opportunity for a more thorough understanding of the similarities and differences between urban and rural populations, contrasting them with the perspectives of experts in the care of the elderly. This triangulation of the views of experts on ageing, in addition to the views of the elderly themselves, helped to provide a more comprehensive understanding of the dimensions of QoL perceived to be important among the elderly.

However, the study findings are limited to the perspectives of elderly individuals living in rural and urban regions of Hai Duong province; and given the social and cultural diversity of this country, national generalization would require careful consideration. Moreover, those living in remote areas may have been missed. Additionally, the results are limited to those who were physically and mentally capable of participating in an IDI or FGD, and as such individuals with disabilities or those who were too sick may not have participated. It is important for future research to explore these missing perspectives, particularly since these individuals represent potentially disadvantaged groups, and needs may differ for these vulnerable populations.

## Conclusions

In this study, six themes including physical, social, financial and economic, environmental psychological, and religious, emerged as the important aspects of QoL among both rural and urban elderly in Vietnam. Person–environment interaction is a major variable in the evaluation of QoL in elderly people. Economic aspects and family ties are also important components of QoL. While older populations share many of the elements of QoL across cultures, this research demonstrates clearly how not only socio-cultural issues, but also geographic issues impinge on understandings of QoL. This research has attempted to bridge this gap in Vietnam by providing insights into both rural and urban perceptions of the dimensions of QoL that are most important to them.

The findings are significant for developing a QoL instrument for the elderly that could be used for comprehensive and long-term outcome evaluation of health promotion intervention programs to this population in Vietnam. The suggested instrument would include subjective assessments of the six themes that were identified as relevant for QoL among elderly Vietnamese, taking into account the differences in level of emphasis among these themes. Visual scales could be used to accommodate for variations in literacy, and recall period would be limited to the present or a short period of time to make the instrument easy for elderly individuals to respond to.

## Footnotes

1 The teachings of the Chinese sage Confucius continue to influence Vietnamese society, with their strong emphasis on moral virtues and normative familial and social relationships that ensure a harmonious society.

## Footnotes

2 Vietnamese culture broadly differentiates between the pagoda, the communal site for formal religious practice (usually Buddhist, or Taoist) and the community temple, more frequently the focus of respect for communal ancestors.
